# Description of *Xiphinema baliense* sp. nov. (Nematoda: Longidoridae), a new member of the *X. americanum*-group from Bali, Indonesia

**DOI:** 10.2478/jofnem-2025-0060

**Published:** 2025-12-29

**Authors:** Franciszek Kornobis, Pablo Castillo, Antonio Archidona-Yuste, Dyah Ayu Oktavianie A. Pratama, Natalia Osten-Sacken, Grażyna Winiszewska, Wiktoria Szydło

**Affiliations:** Institute of Plant Protection - National Research Institute, Władysława Węgorka 20, 60 318 Poznań, Poland; Institute for Sustainable Agriculture (IAS), CSIC, Avenida Menéndez Pidal s/n, 14004 Córdoba, Campus de Excelencia Internacional Agroalimentario, ceiA3,Spain; Faculty of Veterinary Medicine, Universitas Brawijaya, Malang, East Java, 65145, Indonesia; Institute of Veterinary Medicine, Nicolai Copernicus University, Gagarina 7, 87-100 Toruń; Museum and Institute of Zoology, Polish Academy of Sciences, Twarda 51/55, 00–818, Warszawa, Poland; Population Ecology Lab, Faculty of Biology, Adam Mickiewicz University, Poznań, Uniwersytetu Poznańskiego 6, 61-614 Poznań, Poland; Center for Advanced Technologies, Adam Mickiewicz University, Poznań, Uniwersytetu Poznańskiego 10, 61-614 Poznań, Poland

**Keywords:** 28S rDNA, *COI*, D2–D3, detection, ITS, needle nematodes, taxonomy

## Abstract

*Xiphinema baliense* sp. nov., a member of the *X. americanum*-group, *brevicolle*-subgroup, was recovered from the rhizosphere of native vegetation in Bali, Indonesia. Females of the new species are characterized by a moderately long body (2.0–2.4 mm), having a lip region offset by a shallow depression, well-developed odontostyle (106–118 μm), didelphic-amphidelphic equally developed reproductive branches, vulva at 52–55% of body length and a conoid tail (24–31 μm), dorsally convex, ventrally flat with a widely rounded tip. Males were not observed. The alpha-numeric identification codes for the new species are: A5, B23, C2, D2, E3, F-, G2, H2, I2. The new species was morphologically compared with relevant tentative cryptic species in the aforementioned subgroup, including *X. brevicolle, X. himalayense, X. paramonovi, X. primum, X. parabrevicolle*, and *X. purpureum*. Molecular data derived from the D2–D3 expansion segments of 28S rRNA, ITS1, partial 18S rRNA, and *COI* genes were used to reconstruct the phylogenetic relationships of *X. baliense* sp. nov. with related species within the *X. americanum*-group using the Bayesian approach, and the resulting topologies were discussed.

## Introduction

The nematode genus *Xiphinema* Cobb, 1913 comprises ectoparasitic species commonly referred to as dagger nematodes, which parasitize plant roots. It is among the most species-rich genera within the phylum Nematoda Rudolphi, 1808 (Andrássy, 2009), with representatives distributed globally, except Antarctica. Several species within the genus act as vectors for plant nepoviruses (Taylor and Brown, 1997), a role that has led to the inclusion of specific taxa on quarantine pest lists maintained by the European and Mediterranean Plant Protection Organization (OEPP/EPPO, 2024). Moreover, cryptic speciation has been increasingly documented in the genus (e.g., Archidona-Yuste et al., 2016; Jahanshahi Afshar et al., 2019; Cai et al., 2020; Gutiérrez-Gutiérrez et al., 2010; Poureskandarian et al., 2023; Kornobis et al., 2025), underscoring the complexity of its taxonomy. *Xiphinema* is typically divided into two major groups: the *Xiphinema* non-*americanum* group and the *Xiphinema americanum* group. The latter currently comprises approximately 63 described species (Jahanshahi Afshar et al., 2020, 2021; Naghavi et al., 2022; Gu et al., 2023).

Historically, species identification within the genus has relied predominantly on morphological characteristics, leading to the development of identification keys (e.g., Lamberti et al., 2000, 2004). However, species within the *X. americanum* group exhibit pronounced morphological similarity and overlapping morphometric traits, rendering accurate identification challenging, even for experienced taxonomists. A promising solution to this issue lies in the application of integrative taxonomy, which utilizes all available data, primarily morphological features and molecular markers, into a unified identification framework. A critical prerequisite for this approach, however, is the availability of comparative molecular sequences in public databases such as GenBank. Although nematodes of the *X. americanum* group have been studied relatively extensively (at least in comparison to most other soil-inhabiting nematodes and invertebrates), molecular marker data remain unavailable for nearly one-third of the species within this group.

A recently collected soil sample from Bali, Indonesia, yielded a population of *Xiphinema* nematodes. Preliminary morphological and molecular assessments suggest that this population represents a previously undescribed species within the *X. americanum* group. Accordingly, the objectives of this study are i) to characterize the newly recovered population based on morphological traits, morphometric measurements, and molecular markers [including ribosomal regions (D2–D3 expansion domains of 28S rDNA, ITS region, partial 18S rDNA) and the mitochondrial *COI* gene], and formally describe it as a new species; and ii) to elucidate the phylogenetic relationships of this unidentified *Xiphinema* population within the *X. americanum*-group.

## Materials and Methods

### Nematode samples and morphological study

Soil samples containing dagger nematodes were collected from natural habitats near Sanur Town, Bali, Indonesia. Nematodes were extracted using a modified sieving and decanting method (Brown and Boag, 1988). Several specimens were hand-picked and transferred to 1M NaCl for molecular studies, while the remaining ones were heat-killed, fixed in TAF (Courtney et al., 1955), processed to glycerol, and mounted on permanent slides as described by Seinhorst (1959). Identification, measurements, and pictures were conducted using a Zeiss Axioscope microscope equipped with an AxioCam MRc5 camera (Zeiss, Oberkochen, Germany). All other abbreviations used follow the definitions provided by Jairajpuri and Ahmad (1992).

### Molecular characterization

DNA extraction and PCR assays were conducted on single nematodes as described by Castillo et al. (2003). The D2–D3 expansion domains of 28S rRNA were amplified using the D2A (5′-ACAAGTACCGTGAGGGAAAGTTG-3′) and D3B (5′-TCGGAAGGAACCAGCTACTA-3′) primers (De Ley *et al*. 1999). The internal transcribed spacer region 1 (ITS1), which separates the 18S rRNA gene from the 5.8S rRNA gene, was amplified using forward primer 18S (5′-TTGATTACGTCCCTGCCCTTT-3′) (Vrain et al., 1992) and reverse primer rDNA1 5.8S (5′-ACGAGCCGAGTGATCCACCG-3′) (Cherry et al., 1997). A fragment of the mitochondrial *COI* gene was amplified following the protocol of Lazarova et al. (2006), using the primers COIF (5′-GATTTTTTGGKCATCCWGARG-3′) and COIR (5′-CWACATAATAAGTATCATG-3′).

All PCR reactions were performed according to the conditions described by Archidona-Yuste et al. (2016). The amplified products were purified, quantified using ExoSAP-IT (Affymetrix, USB Products, USA), and sequenced directly using a 3130XL Genetic Analyzer (Applied Biosystems, Foster City, CA, USA) with the BigDye Terminator v3.1 Cycle Sequencing Kit (Applied Biosystems) (Tzortzakakis et al., 2014) at the Stab Vida facilities (Caparica, Portugal). Newly obtained sequences were submitted to GenBank under the accession numbers listed in the phylogenetic trees.

### Phylogenetic analyses

The D2–D3 expansion domains of 28S rRNA, ITS1 rRNA, the partial 18S rRNA gene, and *COI* mtDNA sequences from the unidentified *Xiphinema* species population were obtained in this study. These sequences, along with additional sequences of *X. americanum*-group species retrieved from GenBank, were used for phylogenetic analyses. Outgroup taxa for each dataset were selected based on previous studies (Archidona-Yuste et al., 2016; Mobasseri et al., 2019; Gu et al., 2022). Multiple sequence alignments for each locus/gene were performed using the FFT-NS-2 algorithm of MAFFT V.7.450 (Katoh et al., 2019). Sequence alignments were visualized using the BioEdit V. 7.2.5 program (Hall, 1999), and manually edited to trim poorly aligned positions. A light filtering strategy-removing up to 20% of alignment positions-was applied, as recommended by Tan et al. (2015), to maintain phylogenetic accuracy and reduce computation time. This approach was favored over automated filtering methods, which have been shown to compromise single-gene phylogenetic inference (Tan et al., 2015). Phylogenetic analyses of the sequence datasets were conducted using Bayesian inference (BI) implemented in MrBayes v3.1.2 (Ronquist and Huelsenbeck, 2003). The best-fit models of DNA evolution were determined using JModelTest v2.1.7 (Darriba et al., 2012), based on the Akaike information criterion (AIC). The selected models, including base frequencies, proportions of invariable sites, gamma distribution shape parameters, and substitution rates, were incorporated into MrBayes for each dataset. The general time-reversible model with invariable sites and gamma distribution (GTR + I + G) was applied to the D2–D3 expansion domains of 28S rRNA and partial 18S rRNA gene analyses. For ITS1 analysis, the transitional model with invariable sites and gamma distribution (TIM3 + I + G) was selected. In contrast, the one-parameter model with invariable sites and a gamma distribution (TPM3uf + I + G) was used for the partial *COI* gene analysis. Each dataset was analyzed independently using four Markov chains over 10 × 10^6^ generations. Sampling was performed every 100 generations, with two independent runs conducted per dataset. After discarding 30% of the initial samples as burn-in and assessing convergence, the remaining samples were used to reconstruct 50% majority-rule consensus trees. Posterior probabilities (PP) were calculated for all relevant clades. Phylogenetic trees were visualized using FigTree v1.4.4 (Rambaut, 2018).

## Results

### Taxonomy

**Figure 1:**
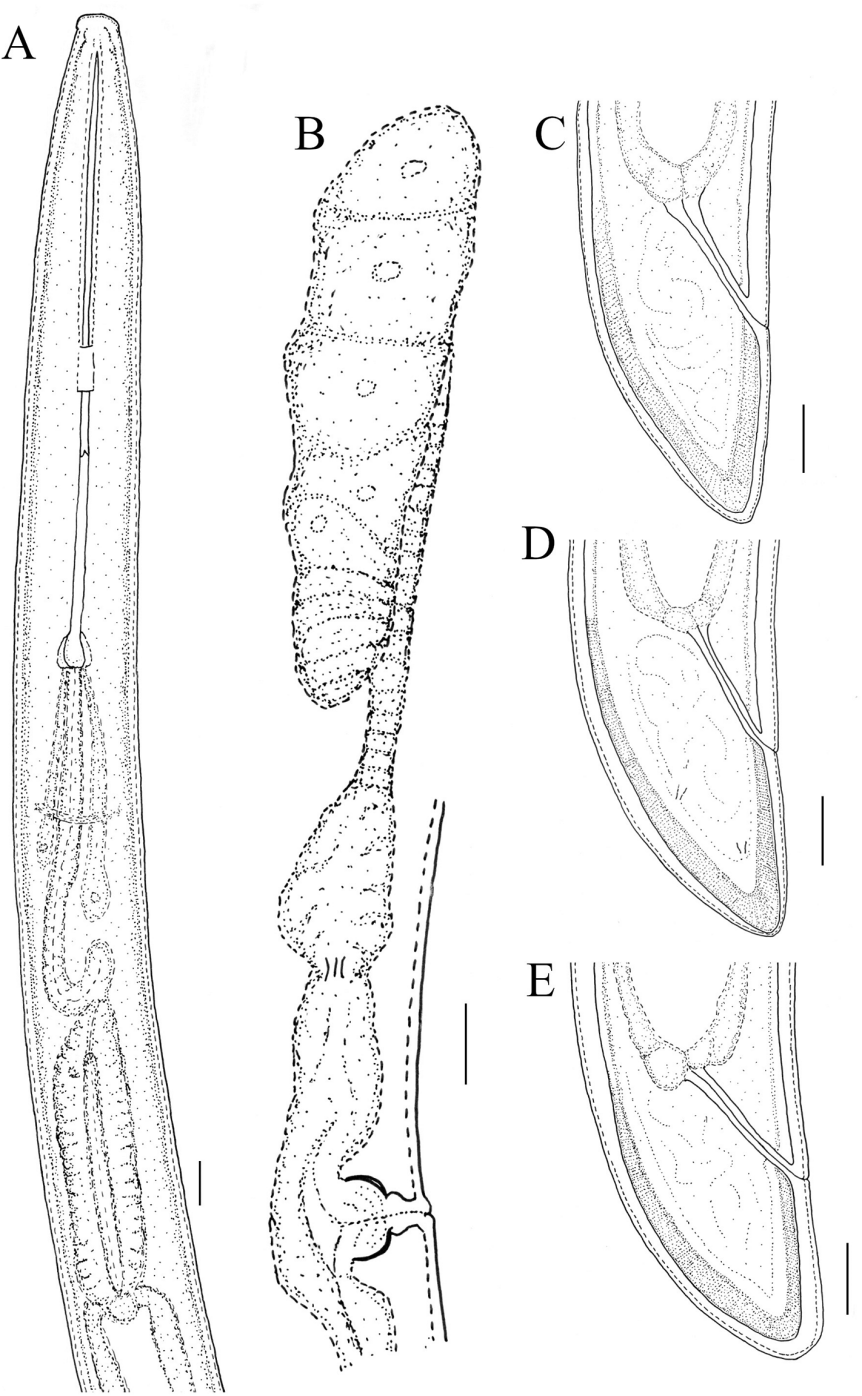
Line drawings of *Xiphinema baliense* sp. nov. (A–E): A) anterior body part; B) anterior genital branch; C–E variation in tails shape. Pictures A–C illustrate the holotype. Scale bar: 10μm.

**Figure 2:**
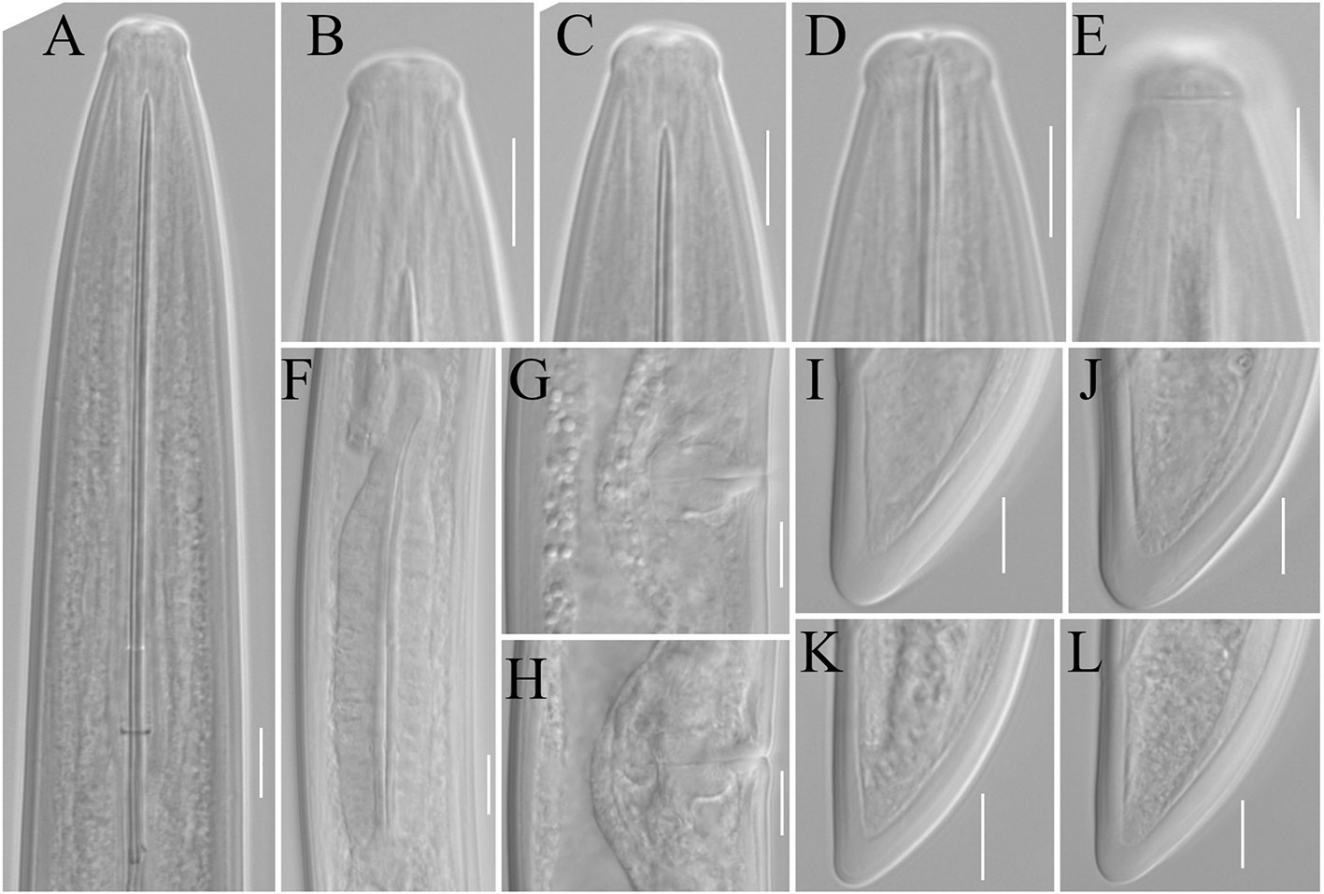
Light microphotographs of *Xiphinema baliense* sp. nov. females (A–L): A) odontostyle region; B–D) variation in lip shape; E) amphidial aperture and fovea; F) pharyngeal bulb; G, H) vulva; I–L) variation in tail shapes. Pictures A, C, F, H, and L illustrate the holotype. Scale bar: 10μm.

**Figure 3:**
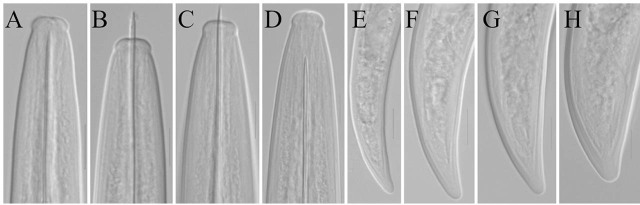
Light microphotographs of *Xiphinema baliense* sp. nov. juveniles (A–H): A–D) anterior end and tails of J1–J4, respectively. Scale bar: 10μm.

*Xiphinema baliense* sp. nov.

(Table 1, Figs. 1–3)

**Table 1:** Morphometrics of *Xiphinema baliense* sp. nov. from Bali. Except body length (mm), all measurements in μm and in format mean ± standard deviation (range).

Trait^*^	**Holotype**	**Holotype + paratype females**	Females for molecular analyses^**^	**J1**	**J2**	**J3**	**J4**
		14	6	3	3	5	6
L	2.2	2.1 ± 0.09 (2.0–2.4)	2.2 ± 0.05 (2.1–2.3)	0.74 (0.7–0.77)	1.0 (0.9–1.2)	1.3 ± 0.05 (1.2–1.3)	1.7 ± 0.09 (1.6–1.9)
a	55.0	52.7 ± 2.1 (49–57)	61.1 ± 3.0 (57–64)	37.7 (35–39)	41.4 (39–43)	43.3 ± 2.52 (40–48)	49.5 ± 4.8 (44–59)
b	6.3	6 ± 0.4 (5.3–6.6)	6.5 ± 0.41 (5.8–6.9)	3.7 (3.3–4.3)	3.7 (3.2–4.3)	4.2 ± 0.24 (3.8–4.5)	4.7 ± 0.1 (4.6–4.8)
c	74.0	77 ± 3.7 (71–86)	71.9 ± 3.4 (68–77)	20.7 (19.7–21)	25.6 (23–30)	34.4 ± 2.48 (30–38)	49.8 ± 3.97 (42–54)
c′	1.2	1.1 ± 0.08 (1–1.24)	1.2 ± 0.11 (1.1–1.3)	2.75 (2.57–3)	2.33 (2.1–2.5)	1.9 ± 0.17 (1.7–2.2)	1.48 ± 0.12 (1.36–1.68)
V	53.0	53 ± 1 (52–55)	52 ± 0.9 (50–53)	-	-	-	-
Odontostyle length	112	113 ± 3.52 (106–118)	110 ± 4.6 (102–115)	50 (43–54)	69.3 (64–72)	84.2 ± 6.6 (76–95)	97.3 ± 4.23 (92–106)
Odontophore length	60	58.7 (55–62)	55.1 ± 2.8 (52–60)	-	-	-	
Total stylet length	172	172 ± 3.7 (165–178)	165.1 ± 6.5 (155–172)	-	-	-	
Replacement odontostyle	-	-	-	61 (51–66)	87.7 (74–98)	99.2 ± 4.4 (95–107)	115.7 ± 3.25 (111–119)
Anterior end to guide ring	103	99.3 ± 2.75 (96–103)	89.2 ± 4.8 (80–94)	-	-	-	
Tail length	30.0	27.9 ± 1.78 (24–31)	30.4 ± 1.59 (29–33)	35.7 (35–36)	39.3 (38–40)	36.6 ± 2.65 (33–40)	34 ± 2.89 (30–39)
Hyaline part of tail length	7.0	6.9 ± 0.83 (6–8)	12.3 ± 0.4 (12–13)	-	-	-	
Width at level of:							
lip region	12.0	12 ± 0.0 (12–12)	11.4 ± 0.4 (11–12)	9 (9–9)	9.3 (9–10)	10.2 ± 0.4 (10–11)	10.7 ± 0.47 (10–11)
vulva or mid-body	40.0	40.6 ± 1.8 (37–45)	35.8 ± 1.9 (34–39)	19.7 (18–21)	24.3 (22–28)	29 ± 2.45 (25–32)	34.3 ± 3.54 (28–39)
Anus	25.0	27.8 ± 1.76 (24–31)	25.4 ± 2.1 (22–28)	13 (12–14)	17 (16–19)	19.2 ± 0.98 (18–20)	23 ± 2.16 (19–26)

* Abbreviations are defined in Jairajpuri and Ahmad (1992).

** These specimens were fixed in 1M NaCl and not TAF, as the rest of the specimens from this table. As the impact of the fixative on the morphometry is well known, measurements from this column cannot be directly compared with the other ones. Additionally, differences in storing conditions (frozen *vs* room temperature) could have affected morphometrics.

*Zoobank registration:* urn:lsid:zoobank.org:act:1DEBBE9D-F36C-45D9-8A6A-BF35BB0BA41F.

### Description

*Female*. Body medium-sized, C-shaped to open spiral, always more coiled posteriorly. Body tapering gradually towards both ends. Cuticle with fine transverse striation in the anterior and posterior body parts, in the mid-body, barely visible, or no striation visible in the light microscope, depending on the specimen. Cuticle 2.0–2.5 μm thick along the body, only on the tail is clearly thicker, 4.0–5.0 μm on both the dorsal and ventral parts, measured at half of the tail length. Lip region flattened to slightly depressed around the oral aperture, laterally rounded, separated from the rest of the body by a shallow depression, 4–5 μm high. The amphidial aperture is at a more or less level of lip constriction, and the amphidial fovea is stirrup-shaped. The guiding ring is rather delicate (at least compared to many other *X. americanum*-group members), double, with the anterior part weakly visible. Guiding sheath approximately 1–3 times longer than wide (when stylet fully retracted). Nerve ring less than one corresponding body width to the basis of the retracted odontophore. Cardia usually rounded, sometimes somewhat conoid. Basal pharyngeal bulb 74–83 x 16–20 μm. Larger dorsal gland (DN) nucleus situated at about 17% of the total bulb length, two smaller ventro-sublateral nuclei (SN) at about the same level and 55% of the bulb length (SN observed in only two specimens). Intestine simple with no particular traits, prerectum often indistinct, rectum 21–24 μm long. The reproductive system is didelphic-amphidelphic, with both branches equally developed. Vulva slit like, vagina perpendicular to body axis, 17–20 μm long or 46–53% of the corresponding body width. *Pars distalis* and *pars proximalis* are 6–8 and 8–12 μm long, respectively. Anterior and posterior uteri 30–48 and 32–46 μm long, respectively. No symbiotic bacteria or sperm cells were observed within the genital tracts under light microscopy. Tail conoid, dorsally convex, ventrally flat with a widely rounded tip. A pair of caudal pores is present on each lateral part of the tail.

#### Male: not found

*Juveniles:* Four juvenile stages present. Body habitus in J1, J to C-shaped, in J2–J4 C-shaped, never spiral. Tail conoid with a rounded tip in all stages, gradually shortening in relation to its width in subsequent stages (c′). Lack of a genital tract and presence of a replacement odontostylet in all stages. Body is smaller than that in females, remaining morphology similar to that of adults. The distinction of stages was based on positions and relative lengths of functional and replacement odontostylets, and body size (Robbins et al. 1996).

#### Type habitat and locality

The new species was recovered from the rhizosphere of *Colocasia* sp., *Monstera* sp., and *Canna* sp., near Sanur town, Bali, Indonesia (coordinates −8.702°, 115.262°).

#### Etymology

The species epithet refers to Bali, Indonesia, the place of species origin.

#### Type material

Holotype female (accession number MIZ PAN NEM 5) and seven female paratypes (MIZ PAN NEM 6–12) deposited at the Museum and Institute of Zoology, Polish Academy of Sciences, Warsaw, Poland; four female paratypes (slides Xbl-01-Xbl-02) at nematode collection of Institute for Sustainable Agriculture (IAS) of Spanish National Research Council (CSIC), Córdoba, Spain; and two females at the USDA Nematode Collection (slide T-8261p).

### Diagnosis and relationships

*Xiphinema baliense* sp. nov. belongs to the *brevicolle*-subgroup of the *Xiphinema americanum*-group. It is primarily characterized by a lip region offset from the body by a shallow depression, and a dorsally convex, ventrally flat conoid tail (24–31 μm long) with a widely rounded tip. Additional diagnostic features include females measuring 2.0–2.4 μm in length, an odontostyle measuring 106–118 μm, the vulva located at 52–55% of body length, four juvenile stages, and the absence of males. The alpha-numeric identification codes of the new species, according to Lamberti et al. (2004), are: A5, B23, C2, D2, E3, F-, G2, H2, I2.

For the differential diagnosis, species selection was based on five diagnostic characters from the key by Lamberti et al. (2004): A5 (odontostyle length between 101–120 μm), B2,3 (vulval position between 51–58%), E3 (body length greater than 2 mm), and H2 (rounded tail). Several closely similar species included in the key, as well as others described later, fulfill these criteria and form tentative cryptic forms of the new species. *Xiphinema baliense* sp. nov. can be distinguished from these species as follows:

From *X. brevicolle* Lordello and Da Costa, 1961 [described initially from Brazil, *X. brevicolle* has since been reported from several continents (Lordello and Da Costa, 1961; Lazarova et al., 2019). However, recent evidence suggests that its accurate distribution is likely restricted to South America (Lazarova et al., 2019), and global records may refer to other species. Comparisons here are based on the original description and data from Lazarova et al. (2019) and Lamberti and Bleve-Zacheo (1979)], by having a longer body (2.0–2.4 *vs*. 1.8–2.2 mm), a slightly longer odontostyle (106–118 *vs*. 85–108 μm), a higher ratio (49–57 *vs*. 37.6–50.1), and a slightly higher c′ ratio (1.0–1.2 *vs*. 0.9–1.1).

From *X. himalayense* Ahmad, Lamberti, Rawat, Agostinelli and Srivastava, 1998, by its offset lip region (vs. continuous), shorter body (2.0–2.4 *vs*. 2.5–2.7 mm), and shorter hyaline tail terminus (6–8 μm *vs.* 8.7–12.0 μm).

From *X. paramonovi* Romanenko, 1981, by a shorter tail (24–31 *vs*. 33–47 μm), shorter hyaline terminus (6–8 μm *vs*. 9–12 μm), and a higher c ratio (71–86 *vs*. 49–68).

From *X. primum* Mobasseri, Hutchinson, Jahanshahi Afshar and Pedram, 2019, by a higher c′ index (1.0–1.2 *vs*. 0.8–1.0) and narrower anal body width (24–31 *vs*. 30–42 μm).

From *X. parabrevicolle* Gutiérrez-Gutiérrez, Cantalapiedra-Navarrete, Decraemer, Vovlas, Prior, Palomares-Rius and Castillo, 2012, by a higher c′ index (1.0–1.2 *vs*. 0.7–0.8) and a greater oral aperture-guiding ring distance (96–106 *vs*. 87–97.5 μm).

From *X. purpureum* Gu, Ye, and Munawar, 2022, by greater oral aperture-guiding ring distance (96–106 *vs*. 83.2–95.2 μm), narrower body (37–45 *vs*. 46.0–52.4 μm), and narrower anal body width (24–31 *vs*. 28.8–33.3 μm).

### Molecular characterization of *Xiphinema baliense* sp. nov.

*Xiphinema baliense* sp. nov. was molecularly characterized using sequences from three ribosomal regions, the D2–D3 expansion domains of 28S rRNA, ITS1 rRNA, and partial 18S rRNA gene, and the mitochondrial gene *COI*. For this species, eight D2–D3 expansion domains sequences (609–776 bp; PX229834-PX229841), eight ITS1 rDNA sequences (756-858 bp; PX229842-PX229849), two partial 18S rRNA gene sequences (959–975 bp; PX229850-PX229851), and four *COI* sequences (293–340 bp; PX220314-PX220317) were obtained. Intraspecific sequence variation was low across both ribosomal and mitochondrial markers: D2–D3 expansion domains of 28S rRNA gene (99.7–100.0% identity, 0–2 bp and 0–1 indels); ITS1 rDNA (98.9–100.0% identity, 0–14 bp and 0 indels); partial 18S rRNA gene with no intraspecific variability detected (100.0 similarity); and *COI* (98.9–100.0% identity, 0–3 bp and 0 indels). D2–D3 expansion domains of 28S rRNA gene of *X. baliense* sp. nov. (PX229834-PX229841) exhibited the following sequence identities: 97.4% to that of *X. santos* Lamberti, Lemos, Agostinelli and D'Addabo, 1993 from Spain (JQ990029), differing by 20 bp and 10 indels (Gutiérrez-Gutiérrez et al., 2012); 97.4% to that if *Xiphinema* sp. from Spain (MH558570), differing by 20 bp and 9 indels (unpublished); 97.4% to that of *X. rivesi* Dalmasso, 1969 from USA (KU680968), differing by 20 bp and 9 indels (Handoo et al., 2016); 97.3% to that of *X. citricolum* Lamberti and Bleve-Zacheo, 1979 from Florida, USA (DQ285668), differing by 21 bp and 10 indels (Gozel et al. 2006); 97.0% to that of *X. georgianum* Lamberti and Bleve-Zacheo, 1979 from Florida, USA (DQ299495), differing by 23 bp and 10 indels (Gozel et al. 2006); and 96.7% to that of *X. oxycaudatum* Lamberti and Bleve-Zacheo, 1979 from South Africa (MK988554), differing by 23 bp and 10 indels (Daramola et al., 2019).

The ITS1 sequences of *X. baliense* sp. nov. (PX229842-PX229849) exhibited the following sequence identities: 86.8–87.0% to those of *X. americanum* Cobb, 1913 from several localities in USA (KF748348-KF748420), differing by 113–116 bp and 66–70 indels (Zasada et al., 2014); 86.9% to that of *X. thornei* Lamberti and Golden, 1986 from Oregon, USA (AY430176), differing by 114 bp and 70 indels (He et al., 2005); 86.8% to that of *X. peruvianum* Lamberti and Bleve-Zacheo, 1979 from Chile (GQ231531), differing by 114 bp and 65 indels (Meza et al., 2011); and 86.8% to that of *X. oxycaudatum* from Taiwan (AY359859), differing by 114 bp and 65 indels (Chen et al. 2005).

The partial 18S rRNA of *X. baliense* sp. nov. (PX229850-PX229851) exhibited high identity (99.9–100.0%) to those of multiple *X. americanum*-group species, including: *X. americanum* from South Africa (AM086684), *X. georgianum* from Florida, USA (AM086688), *X. citricolum* and *X. floridae* Lamberti and Bleve-Zacheo, 1979 from Florida, USA (AM086686 and AM086687), and *Xiphinema* sp. from Maryland, USA (MK292135-MK292136), all of which showed no nucleotide differences (0 bp; 0 indels) (Lazarova et al., 2006; Carta and Li, 2019). Additionally, a single bp difference was observed with that of *X. peruvianum* Lamberti and Bleve-Zacheo, 1979 from Brazil (AY297832) (Oliveira et al., 2004), *X. diffusum* Lamberti and Bleve-Zacheo, 1979 from Australia (AM086685), and *X. taylori* Lamberti, Ciancio, Agostinelli and Coiro, 1992 from Slovakia (AM086675) (Lazarova et al., 2006); all differing by 1 bp and 0 indels.

Finally, the *COI* sequences of *Xiphinema baliense* sp. nov. (PX220314-PX220317) exhibited the following identities: 82.4% to those of *X. rivesi* from Minnesota, USA (KX263102-KX263103), differing by 61 bp and one indel (Orlando et al., 2016); 81.5% to those of *Xiphinema* sp. from California, USA (KX263086-KX263099), differing by 63–67 bp and one indel (Orlando et al., 2016); 80.2–80.4% to those of *X. americanum* from California, USA, and South Africa (KX263047, MN072361), differing by 67 bp and one indel (Orlando et al., 2016; Reighard et al., 2019); 80.2% to that of *X. brevicolle* Lordello and DaCosta, 1961 from Russia (KX263106), differing by 67 bp and one indel (Orlando et al., 2016); and 80.1% to that of *X. peruvianum* from Brazil (AM086712), differing by 58 bp and one indel (Lazarova et al., 2006).

### Phylogenetic analyses

**Figure 4:**
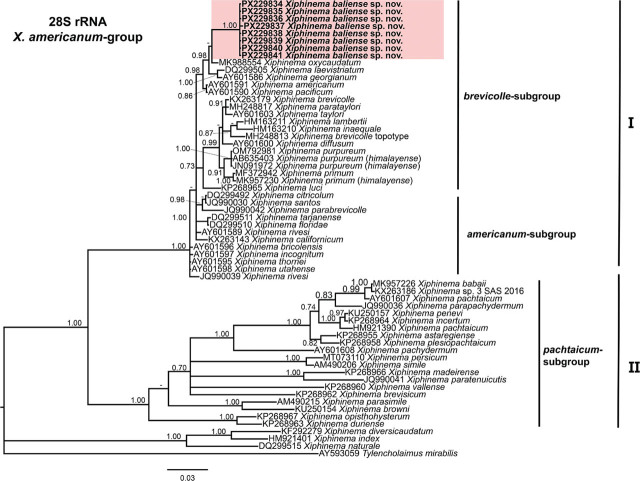
Phylogenetic relationships of *Xiphinema baliense* sp. nov. within *Xiphinema americanum*-group. Bayesian 50% majority rule consensus tree as inferred from D2–D3 expansion segments of 28S rRNA gene sequence alignment under the general time-reversible model with invariable sites and gamma distribution model (GTR + I + G). Posterior probabilities greater than 0.70 are provided for appropriate clades. Newly obtained sequences in this are shown in bold. The scale bar indicates expected changes per site, and the colored boxes indicate clade associations within the *Longidorus* species analyzed in this study.

**Figure 5:**
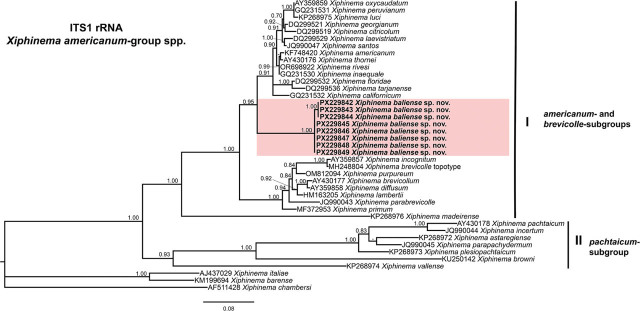
Phylogenetic relationships of *Xiphinema baliense* sp. nov. within *Xiphinema americanum*-group. Bayesian 50% majority rule consensus tree as inferred from ITS1 rRNA gene sequence alignment under the transitional model with invariable sites and gamma distribution (TIM3 + I + G). Posterior probabilities greater than 0.70 are reported for appropriate clades. Newly obtained sequences in this are shown in bold. The scale bar indicates expected changes per site, and the colored boxes indicate clade associations within the *Longidorus* species analyzed in this study.

**Figure 6:**
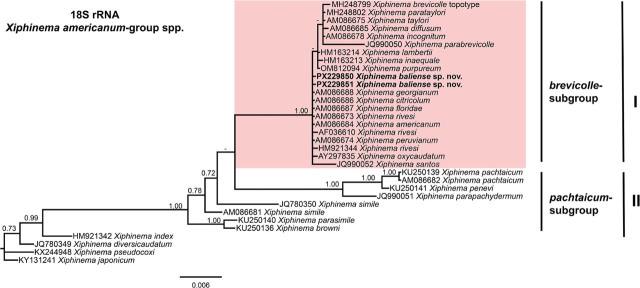
Phylogenetic relationships of *Xiphinema baliense* sp. nov. within *Xiphinema americanum*-group. Bayesian 50% majority rule consensus tree as inferred from 18S rRNA gene sequence alignment under the general time-reversible model with invariable sites and gamma distribution model (GTR + I + G). Posterior probabilities greater than 0.70 are reported for appropriate clades. Newly obtained sequences in this are shown in bold. The scale bar indicates expected changes per site, and the colored boxes indicate the clade association within *Xiphinema americanum*-group species analyzed in this study.

**Figure 7:**
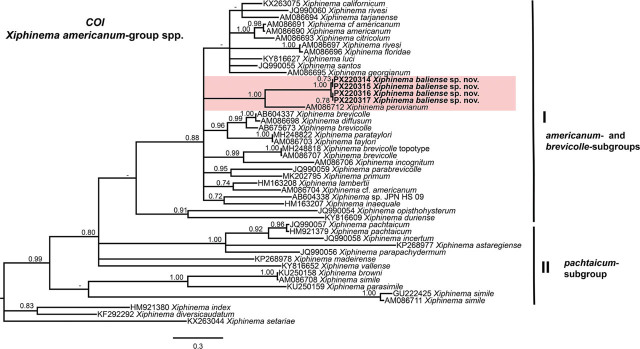
Phylogenetic relationships of *Xiphinema baliense* sp. nov. within *Xiphinema americanum*-group. Bayesian 50% majority rule consensus tree as inferred from COI mtDNA gene sequence alignment under the one-parameter model with invariable sites and gamma distribution model (TPM3uf + I + G). Posterior probabilities greater than 0.70 are reported for appropriate clades. Newly obtained sequences in this are shown in bold. The scale bar indicates expected changes per site, and the colored boxes indicate the clade association within *Xiphinema americanum*-group species analyzed in this study.

Phylogenetic analyses of *Xiphinema americanum*-group species were conducted using BI based on the D2–D3 expansion domains of 28S rRNA, ITS1 rRNA, partial 18S rRNA, and partial *COI* mtDNA sequences (Figs. 4, 5, 6, and 7, respectively). The phylogenetic trees reconstructed from ribosomal and mitochondrial DNA markers included 62, 41, 33, and 47 sequences, with alignments consisting of 777, 932, 1,749, and 392 characters, respectively. The Bayesian 50% majority-rule consensus tree inferred from the D2–D3 expansion domains of 28S rRNA gene is shown in Fig. 4. For this ribosomal marker, included sequences of the *X. americanum*-group species were divided into two well-supported clades (PP = 1.00): Clade I and Clade II (Fig. 4). Clade I include 27 species belonging to the *americanum*-, *brevicolle*-, *lambertii*- and *taylori*-subgroups, as well as all eight sequences of *X. baliense* sp. nov. (PX229834-PX229841), which are clustered together into a distinct, well-supported subclade (PP = 1.00). This subclade is situated within a poorly supported clade (PP = 0.51) alongside sequences of *X. oxycaudatum* (MK988554) from South Africa (Daramola et al., 2019) (Fig. 4). Clade II includes 20 sequences of the species, primarily from the *pachtaicum*-subgroup (Fig. 4).

In the ITS region tree (Fig. 5), phylogenetic analysis revealed that sequences of *X. baliense* sp. nov. (PX229842-PX229849) formed a well-supported clade (PP = 0.95) with those of members of *X. americanum*-group. Sequences of the species belonging to the *pachtaicum*-subgroup clustered in a basal, moderately supported clade (PP = 0.93) (Fig. 5).

In the 18S rRNA phylogenetic analysis (Fig. 6), the two sequences of *X. baliense* sp. nov. (PX229850-PX229851) clustered with 20 other sequences of the species from the *americanum-, brevicolle-, lambertii-,* and *taylori*-subgroups within a well-supported clade (PP = 1.00). However, several subclades within this clade showed weak support (Fig. 6). The relations of remaining sequences of the species in this group were not well resolved, except the subclade comprising sequences of *X. pachtaicum*, *X. penevi*, and *X. pachydermum* (Fig. 6).

Although phylogenetic relationships based on the *COI* gene were not well resolved, the tree has been divided into two main subclades, I and II. The subclade I includes sequences of the species from both the *americanum*- and *brevicolle*- subclades. All four sequences of *X. baliense* sp. nov. (PX220314-PX220317) have clustered together into a distinct, well-supported subclade (PP = 1.00) (Fig. 7).

## Discussion

Lamberti and Ciancio (1993, 1994) proposed a morphological classification of the *X. americanum*-group, dividing its members into five subgroups: *americanum*-, *brevicolle*-, *lambertii*-, *taylori*-, and *pachtaicum*-subgroups. Based on this framework, *X. baliense* sp. nov. is most closely aligned with the *brevicolle*-subgroup. This subgroup is typified by females exhibiting a body length of approximately 1.9 mm, an odontostyle length near 106 μm, and a vulval position (V value) around 53%. In comparison, *X. baliense* sp. nov. shares the odontostyle length and V value (Table 1), although its female body length is notably greater. Despite this difference, phylogenetic analyses (Figs. 4–7) consistently place *X. baliense* sp. nov. within a clade comprising other members of the *X. brevicolle*-subgroup, thereby supporting its taxonomic placement and evolutionary affinity. Close morphological similarity of the species in this group is referred to as cryptic speciation, as already documented (Mobasseri et al., 2019).

Numerous members of the *X. americanum*-group are known to harbor endosymbiotic bacteria within their genital tracts (e.g., Coomans and Claeys, 1998; Vandekerckhove et al., 2000; Palomares-Rius et al., 2016), which are often visible in light microscopy. Subsequent studies have revealed a high degree of specificity between particular nematode and bacterial species, with some authors proposing that bacterial profiles may serve as supplementary criteria for nematode species identification (Orlando et al., 2016; Palomares-Rius et al., 2016). In the present study, no endosymbiotic bacteria were observed (in light microscopy) within the genital tracts of *X. baliense* sp. nov. However, this absence does not contradict previous findings, as Palomares-Rius et al. (2016) demonstrated that molecular techniques can detect endosymbionts that are invisible in light microscopy. Similarly, Gu et al. (2022) reported no observable endosymbionts in *X. purpureum*, another member of the *X. americanum*-group, further underscoring the limitations of microscopy-based detection.

A comprehensive search of the available literature and resources, approved to the best of our knowledge, represents the first report of the occurrence of *Xiphinema* species on Bali Island. However, other *Xiphinema* species may inhabit the island. This hypothesis is supported by Bali's high plant species diversity, which provides a wide array of potential hosts, and by the island's heterogeneous habitats, characterized by variations in humidity, temperature, and other ecological parameters. Such environmental diversity likely fosters numerous microhabitats conducive to nematode colonization. Further reinforcing this assumption is the discovery of *X. baliense* sp. nov. from one of only three soil samples (data not shown), suggesting either a serendipitous encounter or a preliminary glimpse into a broader, yet undocumented, nematode diversity on the island.

Phylogenetic analyses based on D2–D3 and 18S rRNA sequences revealed the affinity of *X. baliense* sp. nov. with sequences of the *brevicolle*-subgroup. Notably, the ITS- and *COI* trees revealed some divergence. These findings underscore the complexity of subgroups within the *X. americanum*-group and highlight the value of multilocus approaches. Overall, the phylogenetic relationships inferred from both ribosomal and mitochondrial markers are congruent with previous studies (Gutierrez-Gutierrez et al., 2012; Archidona-Yuste et al., 2016; Jahanshahi Afshar et al., 2020, 2021; Naghavi et al., 2022; Gu et al., 2023).

In conclusion, this study provides novel insights into the biodiversity of the *X. americanum*-group in a region that remains largely unexplored from a nematological perspective. The integrative data presented here can contribute to future efforts to elucidate the biogeographic origins and evolutionary trajectories of this species complex. Moreover, given that several members of the *X. americanum*-group are recognized as economically significant due to their role as nepovirus vectors, the findings of this study will aid in distinguishing quarantine-relevant taxa from non-vector species, thereby informing pest management and regulatory frameworks.

## References

[j_jofnem-2025-0060_ref_001] Ahmad M., Lamberti F., Rawat V. S., Agostinelli A., Srivastava N. (1998). Two new species within the *Xiphinema americanum*-group (Nematoda, Dorylaimida) from Garhwal Himalayas, India. Nematologia mediterranea.

[j_jofnem-2025-0060_ref_002] Andrássy I., Csuzdi C., Mahunka S. (2009). Free-living nematodes of Hungary (Nematoda errantia).

[j_jofnem-2025-0060_ref_003] Archidona-Yuste A., Navas-Cortés J. A., Cantalapiedra-Navarrete C., Palomares-Rius J. E., Castillo P. (2016). Cryptic diversity and species delimitation in the *Xiphinema americanum*-group complex (Nematoda: Longidoridae) as inferred from morphometrics and molecular markers. Zoological Journal of the Linnean Society.

[j_jofnem-2025-0060_ref_004] Brown D. J. F., Boag B. (1988). An examination of methods used to extract virus-vector nematodes (Nematoda: Longidoridae and Trichodoridae) from soil samples. Nematologia Mediterranea.

[j_jofnem-2025-0060_ref_005] Cai R., Archidona-Yuste A., Cantalapiedra-Navarrete C., Palomares-Rius J. E., Castillo P. (2020). New evidence of cryptic speciation in the family Longidoridae (Nematoda: Dorylaimida). Journal of Zoological Systematics and Evolutionary Research.

[j_jofnem-2025-0060_ref_006] Castillo P., Vovlas N., Subbotin S., Troccoli A. (2003). A new root-knot nematode, *Meloidogyne baetica* n. sp. (Nematoda: Heteroderidae), parasitizing wild olive in Southern Spain. Phytopathology.

[j_jofnem-2025-0060_ref_007] Cherry T., Szalanski A. L., Todd T. C., Powers T. O. (1997). The internal transcribed spacer region of *Belonolaimus* (Nemata: Belonolaimidae). Journal of Nematology.

[j_jofnem-2025-0060_ref_008] Courtney W. D., Polley D., Miller V. L. (1955). TAF, an improved fixative in nematode technique. Plant Disease Reporter.

[j_jofnem-2025-0060_ref_009] Darriba D., Taboada G. L., Doallo R., Posada D. (2012). jModelTest 2: more models, new heuristics and parallel computing. Nature Methods.

[j_jofnem-2025-0060_ref_010] De Ley P., Félix M. A., Frisse L. M., Nadler S. A., Sternberg P. W., Thomas K. W. (1999). Molecular and morphological characterization of two reproductively isolated species with mirror-image anatomy (Nematoda: Cephalobidae). Nematology.

[j_jofnem-2025-0060_ref_011] Gu J., Ye W., Munawar M. (2022). Description of *Xiphinema pupureum* n. sp. (Nematoda: Longidoridae), a new *Xiphinema americanum* group species detected from the rhizosphere of Ilex purpurea from Japan. Nematology.

[j_jofnem-2025-0060_ref_012] Gutiérrez-Gutiérrez C., Cantalapiedra-Navarrete C., Decraemer W., Vovlas N., Prior T., Palomares-Rius J. E., Castillo P. (2012). Phylogeny, diversity, and species delimitation in some species of the *Xiphinema americanum*-group complex (Nematoda: Longidoridae), as inferred from nuclear and mitochondrial DNA sequences and morphology. European Journal of Plant Pathology.

[j_jofnem-2025-0060_ref_013] Gutiérrez-Gutiérrez C., Palomares-Rius J. E., Cantalapiedra-Navarrete C., Landa B. B., Esmenjaud D., Castillo P. (2010). Molecular analysis and comparative morphology to resolve a complex of cryptic *Xiphinema* species. Zoologica Scripta.

[j_jofnem-2025-0060_ref_014] Hall T. A. (1999). BioEdit: a user-friendly biological sequence alignment editor and analysis program for windows 95/98/NT. Nucleic Acids Symposium Series.

[j_jofnem-2025-0060_ref_015] He Y., Subbotin S., Rubtsova T.V., Lamberti F., Brown D.J.F., Moens M. (2005). A molecular phylogenetic approach to Longidoridae (Nematoda: Dorylaimida). Nematology.

[j_jofnem-2025-0060_ref_016] Jahanshahi Afshar F., Shahryari F., Gharibzadeh F., Pourjam E., Pedram M. (2019). Description of *Xiphinema azarbaijanense* n. sp. (Nematoda; Longidoridae) from West Azarbaijan province, northwestern Iran. European Journal of Plant Pathology.

[j_jofnem-2025-0060_ref_017] Jahanshahi Afshar F., Shahryari F., Mokhtassi-Bidgoli A., Castillo P., Fouladvand Z. M., Pedram M. (2020). Occurrence of *Xiphinema santos* Lamberti, Lemos, Agostinelli and D'Addabo 1993 (Nematoda: Longidoridae), a *X. americanum*-group member in Iran. European Journal of Plant Pathology.

[j_jofnem-2025-0060_ref_018] Jahanshahi Afshar F., Pedram M., Mobasseri M. (2021). Description of *Xiphinema persicum* n. sp. (Nematoda: Longidoridae), a *X. americanum*-group species from Iran. European Journal of Plant Pathology.

[j_jofnem-2025-0060_ref_019] Jairajpuri M. S., Ahmad W. (1992). Dorylaimida. Free-living, Predaceous and Plant-Parasitic Nematodes.

[j_jofnem-2025-0060_ref_020] Katoh K., Rozewicki J., Yamada K. D. (2019). MAFFT online service: Multiple sequence alignment, interactive sequence choice and visualization. Brief Bioinformatics.

[j_jofnem-2025-0060_ref_021] Kornobis F., Osten-Sacken N., Winiszewska G., Castillo P. (2024). Cryptic speciation in the nematode family Longidoridae from South America: description of *Xiphinema cryptocostaricense* sp. nov. from Colombia and notes on *X. seinhorsti*. European Journal of Plant Pathology.

[j_jofnem-2025-0060_ref_022] Lamberti F., Bleve-Zacheo T. (1979). Studies on *Xiphinema americanum* sensu lato with descriptions of fifteen new species (Nematoda, Longidoridae). Nematologia Mediterranea.

[j_jofnem-2025-0060_ref_023] Lamberti F., Ciancio A. (1993). Diversity of *Xiphinema americanum-group* species and hierarchical cluster analysis of morphometries. Journal of Nematology.

[j_jofnem-2025-0060_ref_024] Lamberti F., Ciancio A. (1994). The relationship between species within the *Xiphinema americanum*-group (Nematoda: Dorylaimida) 1. EPPO Bulletin.

[j_jofnem-2025-0060_ref_025] Lamberti F., Hockland S., Agosttinelli A., Moens M., Brown D.J.F. (2004). The *Xiphinema americanum* group. III. Keys to species identification. Nematologia Mediterranea.

[j_jofnem-2025-0060_ref_026] Lamberti F., Molinari S., Moens M., Brown D.J. (2000). The *Xiphinema americanum* group. I. Putative species, their geographical occurrence and distribution, and regional polytomous identification keys for the group. Russian Journal of Nematology.

[j_jofnem-2025-0060_ref_027] Lazarova S. S., Malloch G., Oliveira C. M. G., Hübschen J., Neilson R. (2006). Ribosomal and mitochondrial DNA analyses of *Xiphinema americanum*-group populations. Journal of Nematology.

[j_jofnem-2025-0060_ref_028] Lazarova S, Oliveira CM, Prior T, Peneva V, Kumari S (2019). An integrative approach to the study of *Xiphinema brevicolle* Lordello and Da Costa 1961, supports its limited distribution worldwide (Nematoda: Longidoridae). European Journal of Plant Pathology.

[j_jofnem-2025-0060_ref_029] Mobasseri M., Hutchinson M. C., Afshar F. J., Pedram M. (2019). New evidence of nematode-endosymbiont bacteria coevolution based on one new and one known dagger nematode species of *Xiphinema americanum*-group (Nematoda, Longidoridae). PLoS One.

[j_jofnem-2025-0060_ref_030] Naghavi A., Niknam G., Vazifeh N. (2022). A new species of *Xiphinema americanum* group (Nematoda: Longidoridae) from Iran, with additional data on three known species. Systematic Parasitology.

[j_jofnem-2025-0060_ref_031] OEPP/EPPO (2024). European and Mediterranean plant protection organization. EPPO A1, A2 list of pests recommended for regulation as quarantine pests.

[j_jofnem-2025-0060_ref_032] Orlando V., Chitambar J. J., Dong K., Chizhov V. N., Mollov D., Bert W., Subbotin S. A. (2016). Molecular and morphological characterisation of *Xiphinema americanum*-group species (Nematoda: Dorylaimida) from California, USA, and other regions, and co-evolution of bacteria from the genus *Candidatus* Xiphinematobacter with nematodes. Nematology.

[j_jofnem-2025-0060_ref_033] Palomares-Rius J.E., Archidona-Yuste A., Cantalapiedra-Navarrete C., Prieto P., Castillo P. (2016). Molecular diversity of bacterial endosymbionts associated with dagger nematodes of the genus *Xiphinema* (Nematoda: Longidoridae) reveals a high degree of phylogenetic congruence with their host. Molecular Ecology.

[j_jofnem-2025-0060_ref_034] Poureskandarian M. A., Pourjam E., Pedram M. (2023). New evidence on cryptic speciation in soil-inhabiting nematodes, with morphological and molecular characterisation of *Xiphinema sangesarense* n. sp. (Dorylaimida: Longidoridae) from north-central mountains of Iran. Nematology.

[j_jofnem-2025-0060_ref_035] Rambaut A. (2014). FigTree v1.4.2, A Graphical Viewer of Phylogenetic Trees.

[j_jofnem-2025-0060_ref_036] Robbins R.T., Brown D.J.F., Halbrendt J.M., Vrain T.C. (1996). Compendium of juvenile stages of *Xiphinema* species (Nematoda: Longidoridae). Russian Journal of Nematology.

[j_jofnem-2025-0060_ref_037] Romanenko N.D. (1981). Discovery of a new species of nematode *X. paramonovi* n. sp. (Nematoda: Longidoridae) in the territory of the Soviet Union.

[j_jofnem-2025-0060_ref_038] Ronquist F., Huelsenbeck J. P. (2003). MRBAYES 3: Bayesian phylogenetic inference under mixed models. Bioinformatics.

[j_jofnem-2025-0060_ref_039] Rudolphi C.A. (1808). Enterozoorum Sive Vermium Intestinalium Historia Naturalis.

[j_jofnem-2025-0060_ref_040] Seinhorst J.W. (1959). A rapid method for the transfer of nematodes from fixative to anhydrous glycerine. Nematologica.

[j_jofnem-2025-0060_ref_041] Tan G., Muffato M., Ledergerber C., Herrero J., Goldman N., Gil M., Dessimoz C. (2015). Current methods for automated filtering of multiple sequence alignments frequently worsen single-gene phylogenetic inference. Systematic Biology.

[j_jofnem-2025-0060_ref_042] Taylor C.A., Brown D.J.F. (1997). Nematode Vectors of Plant Viruses.

[j_jofnem-2025-0060_ref_043] Tzortzakakis E. A., Archidona-Yuste A., Cantalapiedra-Navarrete C., Nasiou E., Lazanaki M. S., Kabourakis E. A., Palomares-Rius J. E., Castillo P. (2014). Integrative diagnosis and molecular phylogeny of dagger and needle nematodes of olives and grapevines in the island of Crete, Greece, with description of *Xiphinema cretense* n. sp. (Nematoda, Longidoridae). European Journal of Plant Pathology.

[j_jofnem-2025-0060_ref_044] Vandekerckhove T. T., Willems A., Gillis M., Coomans A. (2000). Occurrence of novel verrucomicrobial species, endosymbiotic and associated with parthenogenesis in *Xiphinema americanum*-group species (Nematoda, Longidoridae). International Journal of Systematic and Evolutionary Microbiology.

[j_jofnem-2025-0060_ref_045] Vrain T. C., Wakarchuk D. A., Levesque A. C., Hamilton R. I. (1992). Intraspecific rDNA restriction fragment length polymorphism in the *Xiphinema americanum* group. Fundamental and Applied Nematology.

